# ESTIMATING THE ECONOMIC VALUE OF FAMILY CAREGIVING AT A NATIONAL AND STATE LEVEL

**DOI:** 10.1093/geroni/igad104.0793

**Published:** 2023-12-21

**Authors:** Selena Caldera, Susan Reinhard, Ari Houser, Rita Choula

**Affiliations:** AARP, Washington, District of Columbia, United States; AARP, Washington, District of Columbia, United States; AARP Public Policy Institute, Washington, District of Columbia, United States; AARP, Washington, District of Columbia, United States

## Abstract

The 2023 update to the Valuing the Invaluable series estimates the economic value of unpaid, family caregiving in America at $600 billion in 2021. This is the latest estimate in almost 20 years of work tracking the economic value of family caregiving in the U.S. The Valuing the Invaluable series of reports uses a meta-analysis approach drawing on various surveys of family caregivers to produce estimates at the state and national levels of the numbers of caregivers, the hours of care they provided, the average economic value of that care per hour, and the total economic value of all caregiving provided in that year. This presentation will detail the methodology for this meta-analysis, the 2021 national estimate of the economic value of caregiving, and variation in state-level estimates of the economic value of caregiving. While this estimated economic value of caregiving seeks to be comprehensive, it does not account for opportunity costs tied to forgoing work or scaling back work because of caregiving, out-of-pocket costs of caregivers, or foregone retirement savings of caregivers. These limitations will be discussed and the direction they offer for future work understanding the full value of unpaid caregiving contributions to the LTSS system and the state and federal budgets.

